# Impact of Palliative Gastrojejunostomy Type on Survival in Patients with Unresectable Pancreatic Head Cancer: A Retrospective Analysis

**DOI:** 10.3390/biomedicines14020326

**Published:** 2026-01-31

**Authors:** Oliwia Grząsiak-Kraj, Tomasz Kraj, Janusz Strzelczyk

**Affiliations:** 1 Department of General and Transplant Surgery, Medical University of Lodz, 90-419 Lodz, Poland; janusz.strzelczyk@umed.lodz.pl; 2 Department of Vascular Surgery and Angiology, Independent Public Healthcare Institution of the Ministry of the Interior and Administration in Łódź, 42 Północna Street, 91-425 Lodz, Poland

**Keywords:** pancreatic cancer, palliative surgery, gastrojejunostomy, Roux-en-Y, omega loop gastrojejunostomy, overall survival

## Abstract

Gastrojejunostomy (GJ) is used as a palliative procedure in patients with inoperable pancreatic head cancer. While its primary goal is to relieve obstruction, clinical observations suggest the type of reconstruction may influence survival. **Objectives**: The objective of this study was to compare overall survival in patients undergoing different types of palliative gastrojejunostomy. **Methods**: A retrospective analysis was performed on 240 patients with inoperable pancreatic cancer. Patients were divided into three groups: Roux-en-Y (*n* = 186), Omega loop with Braun anastomosis (*n* = 36), and simple Omega loop (*n* = 18). A multivariable Cox proportional hazards model was used to assess the risk of death, adjusting for age and sex. **Results**: In the multivariable analysis, the Roux-en-Y anastomosis was associated with a significantly lower risk of death compared to the simple Omega loop (HR = 0.47; 95% CI: 0.28–0.79; *p* = 0.004). Similarly, the Omega loop with Braun anastomosis demonstrated a significant survival benefit compared to the simple Omega loop (HR = 0.50; 95% CI: 0.27–0.93; *p* = 0.029). Age was a significant independent predictor of mortality. **Conclusions**: The type of gastrojejunostomy significantly influences survival in patients with advanced pancreatic cancer. Both Roux-en-Y and Omega with Braun anastomosis offer superior survival outcomes compared to simple Omega loop gastrojejunostomy. These benefits may be attributable to complex metabolic and hormonal mechanisms, which warrant further investigation in prospective studies.

## 1. Introduction

Pancreatic cancer is one of the most aggressive malignancies of the gastrointestinal tract, with a 5-year survival rate below 10%, mainly due to late diagnosis and limited resectability at the time of presentation [1,2]. Tumours located in the pancreatic head frequently lead to gastrointestinal obstruction, resulting in deterioration of nutritional status, reduced quality of life, and accelerated decline in the patient’s general condition [3]. In patients with unresectable disease, palliative interventions are therefore essential and are primarily aimed at symptom relief and maintenance of adequate nutritional status [4].

Gastrojejunostomy (GJ) is a well-established surgical method for the treatment of malignant gastric outlet obstruction, performed both therapeutically and prophylactically during exploratory laparotomy in patients operated on for pancreatic cancer [5,6]. Initially, the primary goal of GJ was to ensure alimentary tract continuity and to prevent complications related to obstruction. However, increasing evidence indicates that gastrointestinal reconstruction may exert systemic metabolic, hormonal, and immunological effects that can potentially influence long-term outcomes, including survival [6,7,8].

Different types of gastrojejunostomy, such as simple loop (omega) GJ, Roux-en-Y reconstruction, and omega loop with Braun enteroenterostomy, differ substantially in terms of intestinal configuration, bile flow, and alimentary transit [9]. These anatomical differences may affect enteroendocrine signalling, gut microbiota composition, glucose metabolism, and inflammatory pathways, all of which are recognised as factors involved in cancer progression and patient prognosis.

Although Roux-en-Y reconstruction has been extensively studied in the context of bariatric surgery and metabolic disorders, its potential impact on survival in patients undergoing palliative surgery for pancreatic cancer remains poorly understood. Direct comparative studies evaluating survival according to the type of gastrojejunostomy performed in patients with advanced pancreatic cancer are scarce.

In light of this gap in the literature, the aim of the present study was to compare overall survival in patients with unresectable pancreatic head cancer undergoing different types of palliative gastrojejunostomy.

## 2. Materials and Methods

A retrospective analysis was performed on a cohort of patients who underwent palliative gastrojejunostomy for inoperable pancreatic head cancer at a single centre between 2010–2024. The inclusion criteria were: histologically confirmed pancreatic ductal adenocarcinoma, unresectable disease determined intraoperatively, and qualification for palliative gastrojejunostomy due to duodenal obstruction or prophylaxis of obstruction. Patients were categorized into three groups based on the surgical technique performed:

1. Roux-en-Y gastrojejunostomy (Group R),
2. Omega loop gastrojejunostomy with Braun enteroenterostomy (Group O + B),
3. Simple Omega loop gastrojejunostomy (Group O).

The choice of surgical technique was determined by the operating surgeon’s preference and intraoperative anatomical conditions.

Statistical Analysis: Survival analysis was performed using the Kaplan–Meier method and compared using the log-rank test. Overall survival (OS) was defined as the time from the date of surgery to the date of death. Data for living patients were censored at the date of the last follow-up (5 November 2024). To identify independent predictors of mortality, a multivariable Cox proportional hazards regression model was employed, adjusting for age and sex. Results were reported as Hazard Ratios (HR) with 95% Confidence Intervals (95% CI). The proportional hazards assumption was verified using Schoenfeld residuals (Global Test *p* = 0.19), confirming the validity of the model over the entire follow-up period. Due to the retrospective nature of the study and the inclusion of all available eligible patients, no formal a priori sample size calculation was performed. Statistical significance was set at *p* < 0.05. All analyses were conducted using R software version 4.3.1.

## 3. Results

The study enrolled a total of 240 patients. The study population consisted of 121 women (50.4%) and 119 men (49.6%), with a mean age of 69.4 years (range: 28–89; median: 71 years). The majority of patients underwent Roux-en-Y anastomosis (*n* = 186; 77.5%), followed by Omega loop with Braun anastomosis (*n* = 36; 15.0%) and simple Omega loop gastrojejunostomy (*n* = 18; 7.5%).

**Figure 1:**
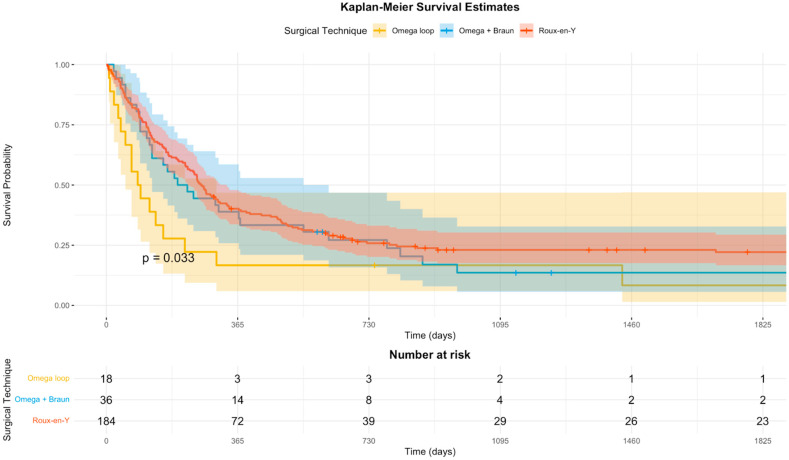
Kaplan–Meier survival estimates stratified by surgical technique. The curves demonstrate the overall survival probability for patients undergoing simple Omega loop (yellow), Omega loop with Braun enteroenterostomy (blue), and Roux-en-Y gastrojejunostomy (red). The log-rank test indicates a statistically significant difference between the curves (*p* = 0.033). Shaded areas represent 95% confidence intervals. The x-axis is truncated at 1800 days for better visualization of the early follow-up period.

Kaplan–Meier survival estimates for the three study groups are presented in Figure 1. Multivariable Cox proportional hazards regression analysis was performed to identify independent predictors of mortality over the entire follow-up period, adjusting for age and sex. Age at the time of surgery was a significant predictor of survival, with each additional year increasing the risk of death by approximately 5% (HR = 1.05; 95% CI: 1.03–1.06; *p* < 0.001). Sex was not a statistically significant predictor (*p* = 0.25).

**Figure 2:**
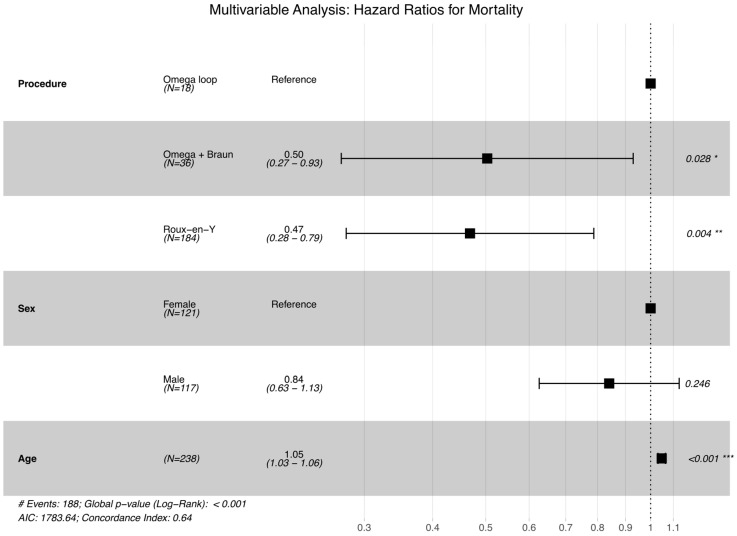
Forest plot visualizing the results of the multivariable Cox proportional hazards regression analysis. The plot displays Hazard Ratios (HR) with corresponding 95% Confidence Intervals (CI) for mortality. The model was adjusted for age and sex. The simple Omega loop serves as the reference group. Both Roux-en-Y and Omega with Braun anastomosis show a statistically significant reduction in mortality risk (shifted to the left of the reference line), while older age is associated with increased risk. Significance codes: * *p* < 0.05, ** *p* < 0.01, *** *p* < 0.001. The symbol ‘#’ indicates the total number of patients.

Regarding the surgical technique, using the simple Omega loop as the reference group, the Roux-en-Y gastric bypass was associated with a significant 53% reduction in the risk of mortality (HR = 0.47; 95% CI: 0.28–0.79; *p* = 0.004). The Omega loop with Braun enteroenterostomy (O + B) also demonstrated a statistically significant survival benefit compared to the reference group, with a 50% risk reduction (HR = 0.50; 95% CI: 0.27–0.93; *p* = 0.029) (Figure 2).

## 4. Discussion

In this retrospective study of 240 patients who underwent palliative gastrojejunostomy due to advanced pancreatic cancer, significant variations in overall survival were observed, depending on the surgical anastomosis technique employed. These findings highlight the importance of selecting the type of gastrojejunostomy even in the palliative setting, where the primary goal is not to prolong survival but to improve patient comfort.

The study demonstrated that palliative Roux-en-Y anastomosis was associated with a 53% reduction in the risk of death as compared to simple gastrojejunostomy (HR = 0.47, *p* = 0.004). The findings of the study may imply that the gastrointestinal tract reconfiguration, entailing the formation of two intestinal loops, i.e., enzymatic and alimentary ones, may enhance gastrointestinal function and reduce the risk of complications, thus extending survival in patients with advanced pancreatic head cancer [5,6].

Interestingly, the Omega loop with Braun enteroenterostomy also exhibited a statistically significant improvement in survival as compared to the simple Omega loop (HR = 0.50, *p* = 0.029). The study revealed no statistically significant difference between the Roux-en-Y and Omega with Braun groups, which may indicate that additional Braun enteroenterostomy compensated for the disadvantages of simple gastrojejunostomy [6].

The effect provided by the Roux-en-Y and Omega with Braun techniques may result from a number of changes that are likely to occur. The differences affect food passage, hormonal profile and gut microbiota, which, in turn, impacts metabolic status and potential survival. Although further verification of the findings in prospective studies is required, several pathophysiological hypotheses may provide a rationale for the received results.

The findings suggest that the use of gastrojejunostomy (GJ) in patients with inoperable pancreatic head cancer may have a favourable impact on survival, the conclusion that is also corroborated by other literature reports [7].

One of the pivotal mechanisms in the process is the gut–brain axis modification. Gastrojejunostomy causes bypassing of the duodenum and initial section of the small intestine, thereby accelerating food passage to further sections of the small intestine. Consequently, L-type enteroendocrine cells are activated, producing incretin hormones, predominantly GLP-1 (glucagon-like peptide-1) and PYY (peptide YY) [7,8,9,10,11].

Numerous studies have demonstrated that PYY secretion significantly increases after gastric bypass is performed using the Roux-en-Y method [12,13]. There is evidence to suggest that a similar phenomenon also occurs after palliative Roux-en-Y anastomosis, as demonstrated in this study.

PYY not only regulates peristalsis, digestive secretion and appetite, but it also has immunological and anti-proliferative effects. It inhibits the release of pro-inflammatory cytokines and amylase by interacting with transcription factors at the acinar level [7]. Importantly, PYY may act as a potent inhibitor of pancreatic cancer cell proliferation in vitro, with data indicating a cell growth reduction of approximately 24–25% [14]. Consequently, Roux-en-Y operations, resulting in elevated PYY concentrations, may theoretically slow tumour progression and improve patient survival.

GLP-1, the other hormone secreted by L-cells, shows pleiotropic properties. Those include anti-inflammatory effects (inhibiting, among others, TNF-α and IL-6), improvements in mitochondrial function and reductions in oxidative stress [10,15]. Preclinical studies have also indicated its potential to exhibit anticancer properties; however, the findings remain equivocal [8].

Furthermore, gastrojejunostomy may exert an influence on the gut microbiota composition. Post-surgical alterations in intestinal transit and environment (pH, bile acid and enzyme availability) may foster the proliferation of bacteria such as *Akkermansia muciniphila* and *Faecalibacterium prausnitzii*, which produce short-chain fatty acids (SCFAs) [16,17]. SCFAs have anti-inflammatory and immunomodulatory effects, with the potential to influence cancer cell proliferation by activating GPR41 and GPR43 receptors [16].

The beneficial influence on glucose metabolism is also significant. GLP-1 and altered microbiota may improve insulin sensitivity and reduce glucose levels [9,15,18,19], a significant finding given the established correlation between hyperglycaemia and a poorer prognosis in patients with pancreatic cancer [18,19].

Furthermore, the mechanical aspect of GJ cannot be disregarded. The operation prevents duodenal obstruction, enabling continuation of oral nutrition and minimising the need for drains and tubes, which directly impacts patient quality of life and, potentially, patient survival [3,5].

In summary, the mechanisms through which gastrojejunostomy exerts its beneficial effects on survival are complex and probably synergistic. They include hormonal effects (PYY, GLP-1), influence on the microbiota, improved metabolism and mechanical factors. Further prospective studies are required, incorporating hormonal and microbiota biomarker evaluation, in order to ascertain the causality of the observed relationships.

Our study has several limitations. Its retrospective design is associated with a risk of selection bias. There was a considerable imbalance in patient numbers between the treatment groups (186 vs. 36 vs. 18). Specifically, the small sample size of the simple Omega loop group serves as a limitation; however, the high number of events (188 deaths) in the cohort provided sufficient statistical power to detect significant differences in the multivariable model. Additionally, while all patients qualified for palliative chemotherapy, detailed data on completion rates and specific regimens were not uniformly available due to the retrospective nature of the study. Finally, the study did not directly assess changes in incretin hormone levels or gut microbiota composition, and the proposed mechanisms are based on data derived from the existing literature.

## 5. Conclusions

The type of gastrojejunostomy may have a multidirectional effect on the survival of patients with unresectable pancreatic cancer, affecting metabolic, hormonal, microbiotic and mechanical processes. Whilst the obtained results are encouraging, further validation is required through prospective studies incorporating hormonal and microbiological biomarker evaluation. The obtained results may be relevant for surgical decision-making in patients with unresectable pancreatic cancer and indicate the need for further research.

## Data Availability

The data presented in this study are available on request from the corresponding author. The data are not publicly available due to privacy or ethical restrictions.

## Abbreviations

The following abbreviations are used in this manuscript:

GJ	Gastrojejunostomy
OS	Overall survival
HR	Hazard Ratios
CI	Confidence Intervals
GLP-1	Glucagon-like peptide-1
PYY	Peptide YY
SCFAs	short-chain fatty acids
